# Liver Transplantation for Acute Hepatic Failure Following Intentional Iron Overdose

**DOI:** 10.7759/cureus.48392

**Published:** 2023-11-06

**Authors:** Rosário Eça, Sofia Ferreira, Judit Gandara, Helena Pessegueiro, Jorge Daniel

**Affiliations:** 1 Medicina Interna, Centro Hospitalar Lisboa Central, Lisboa, PRT; 2 Unidade de Transplantação Hepática e Pancreática, Centro Hospitalar Universitário do Porto, Porto, PRT

**Keywords:** hepatotoxicity, suicide attempt, liver transplant, acute liver failure, iron intoxication

## Abstract

The majority of acute iron toxicity cases occur in young children from accidental ingestion. In adults, iron poisoning is rare and mostly due to intentional ingestion. Physicians, particularly those who do not routinely treat pediatric patients, are often unfamiliar with the clinical manifestation of iron poisoning, its management, and its potential for multiple organ failure, especially liver damage. Severe acute hepatotoxicity treated with liver transplantation is rare in adults, with very limited published literature. We report a case of a severe iron tablet overdose with suicidal intent that progressed to fulminant hepatic failure despite medical treatment, ultimately treated with liver transplantation.

## Introduction

Acute iron toxicity after oral intake is more common in children and is one of the leading causes of accidental poisoning deaths in pediatric patients. In adults, iron poisoning is more commonly associated with suicide attempts, most frequently in young adult females [1]. 

Iron is an essential element for normal cell function, which in excess is also highly cytotoxic. As there is no process for iron elimination, gastrointestinal iron absorption is a carefully regulated process [2]. The dose makes the poison; consequently, side effects of iron overdose are mostly dependent on the quantity of elemental iron ingested per body weight. Symptoms are usually absent for doses under 20 mg/Kg, and between 20 and 60 mg/Kg, mild to moderate gastrointestinal symptoms are to be expected. Ingestions greater than 60 mg/kg pose an increased risk for serious systemic toxicity and death [1,2]. The clinical course of iron toxicity is classically divided into five stages, with earlier stages (stages 1 and 2) representing local gastrointestinal corrosive effects and the latter stages prevailing with systemic toxicity (stages 3 and 4) and chronic sequelae (stage 5) [1-3].

Treatment of mild to moderate iron poisoning is mainly supportive as, in most cases, no serious consequences occur. Iron chelating therapy with deferoxamine is the standard of care for patients with systemic toxicity. Liver transplantation as a lifesaving treatment for liver failure can be considered, but with few cases published, mostly in the pediatric age group and with variable outcomes [4,5].

We describe a clinical case of a woman transferred to our unit with severe iron-induced-hepatic failure successfully treated with emergent liver transplantation. This case serves to emphasize the importance of identifying patients at risk for hepatotoxicity and the potentially lifesaving role of liver transplantation in iron-induced fulminant hepatic failure.

## Case presentation

A 38-year-old woman with a history of depression and obesity presented to the emergency department of a secondary hospital 4 hours after the ingestion of around 90 ferrous sulfate pills (9,45 g of elemental iron, corresponding to 172 mg/kg body weight). At presentation, the patient had gastrointestinal complaints (nausea, vomiting, and abdominal pain), was lethargic but arousable and there was no evidence of neurological deficits or hemodynamic instability. Initial laboratory testing was mostly normal other than elevated lactate levels (3.1 mmol/L) and free iron (1045 μg/dL). Serum toxicology was negative for salicylates, acetaminophen, ethanol, or drugs. She underwent gastric lavage along with supportive care. Though she was clinically stable, due to concerning free blood iron levels and in anticipation of a possible clinical deterioration, she was transferred to the intensive care unit for close monitoring, and intravenous deferoxamine was initiated, followed by empiric N-acetylcysteine once liver injury became apparent as recommended by Poison Control Center.

On the following day, she became progressively hemodynamic unstable and encephalopathic. Laboratory tests (Table 1) showed marked elevation in serum aminotransferases levels (aspartate aminotransferase 8674 UI/L and alanine aminotransferase 7877 UI/L) and international normalized ratio (INR 9,3), slightly elevated total bilirubin and abnormal acid-base parameters (pH 7,28), heralding acute liver failure and metabolic acidosis.

**Table 1:** Laboratory test on day two after admission. Legend: (H) – high; (L) – Low.

	Value	Reference range
Iron (ug/dL)	1045 (H)	33-193 (ug/dL)
pH	7.289	7.35-7.45
Lactic acid (mmol/L)	3.1 (H)	0.5-1.5 (mmol/L)
Bicarbonate (mEq/L)	13.9 (L)	22-28 (mEq/L)
International Normalized Ratio	9.3 (H)	0.8-1.2
Prothrombin time (s)	12.8 (H)	9.4-12.5 (s)
Aspartate aminotransferase(IU/L)	8674 (H)	10-30 (IU/L)
Alanine aminotransferase (IU/L)	7877 (H)	10-36 (IU/L)
Unconjugated bilirrubin (mg/dL)	0.92	0.0-1.0 (mg/dL)
Conjugated bilirrubina (mg/dL)	1,42 (H)	0.0-0.3 (mg/dL)
Albumin (g/dL)	2.90 (L)	3.5-5.0 (g/dL)
Creatinine (mg/dL)	3.69 (H)	0.5-0.9 (mg/dL)

Hemodialysis was initiated in an attempt to control metabolic acidosis, but her clinical condition was rapidly deteriorating. At the time she was transferred to our hospital, a liver transplant center, her Model of End Stage Liver Disease (MELD) score was 35. Considering the patient’s young age and fulminant liver failure, a lifesaving liver transplant was considered and authorized at our liver transplant multidisciplinary consult. All supportive care was continued, and three days later she underwent a successful orthotopic liver transplant. Post-operation was uneventful, with improving clinical signs and liver function parameters. The patient was discharged from our liver transplant unit three months after admission with normal graft function and normal liver and renal function.

## Discussion

Iron is a readily available over-the-counter supplement with the potential for significant deleterious effects, whether by intentional or unintentional use. The most common iron formulation is 325 mg ferrous sulfate, which contains 20% elemental iron per tablet. Overdose in adults is infrequent and occurs typically as a result of a suicide attempt.

Iron promotes direct mucosa irritation and at the intracellular level favors free radical production, oxidative damage, hinders oxidative phosphorylation, and ultimately causes cell death [2]. Clinically, iron toxicity is divided into five stages based on symptoms and clinical manifestations that reflect the two most important effects of iron toxicity: gastrointestinal and circulatory/liver injury [1-3]. In stage I (0-6 h after ingestion), the patient exhibits early gastrointestinal mucosal damage and symptoms include abdominal pain, vomiting, diarrhea, hematemesis, and hematochezia (from mucosal necrosis). In stage II (6-24h after ingestion) there is an apparent latent phase and symptoms may subside; Stage III (12-72h after ingestion) is characterized by coagulopathy, metabolic acidosis, hemodynamic instability, and ultimately shock. Stage IV (12-96h) is characterized by hepatotoxicity and signs of acute liver failure. Stage V (3-6 weeks) occurs after the acute phase, when patients experience symptoms of gastrointestinal obstruction due to strictures, most commonly gastric outlet obstruction [1,3]. Of note, progression through these phases may occur rapidly in large oral overdoses, and death from iron poisoning is usually from shock or acute liver failure [1]. 

Iron-induced-hepatotoxicity is thought to be a dose-related damage as concluded by Robertson and Tenenbein in a retrospective study of 73 patients with iron intoxication. In a subgroup with severe hepatotoxicity (n=9), all patients exhibited serum iron concentration above 1000 μg/dL within the first 12h, thus associating higher doses with an increased risk of clinically important hepatotoxicity [6].

Acute liver failure (ALF) is defined as a rapid deterioration of the liver function associated with hepatic encephalopathy within 26 weeks of jaundice in a patient with no previous known hepatopathy [7]. Drug-induced ALF accounts for approximately 50% of the cases in developed countries, especially with paracetamol. Without liver transplants, patients with ALF have a very high mortality rate between 80-85% [7,8]. Several prognostic scores have been used to predict the outcome in ALF patients and identify patients with a high likelihood of mortality that would benefit from a liver transplant. The King’s College Criteria developed in 1989 is the most widely applied prognostic criteria, having shown high specificity but moderate sensibility [7]. To predict patients with ALF at high risk of mortality, it uses major criteria (prothrombin time >100 s, INR > 6.5) or a combination of at least three minor criteria (Age <10 or >40 years; etiology non-A/non-B hepatitis or drug-induced, duration of jaundice to hepatic encephalopathy >7 days, prothrombin time >50 (INR > 3.5) and serum bilirubin >300 mol/L). Initially used for short-term mortality among patients with cirrhosis, the MELD score has also been applied in an ALF context, using a composite formula based on three laboratory parameters: total serum bilirubin, serum creatinine, and INR. Predictive cutoffs vary across studies, but some authors have suggested scores higher than 30 indicative of the need for urgent liver transplant in non-paracetamol ALF [8]. Our patient had fulfilment of both King’s College and MELD score criteria for lifesaving liver transplantation.

ALF secondary to iron poisoning is very rare in clinical practice, so experience across centres is limited. The specific timing and indications for liver transplant following severe iron poisoning are uncertain, ultimately relying on the assessment and commitment of the liver multidisciplinary team. Psychiatric diseases can be a relative contraindication for liver transplant (American Association for the Study of Liver Diseases) and liver transplant candidates often have comorbid psychiatric disorders, which are particularly sensitive in the context of a suicide attempt as in the case of our patient [9]. Instead of weeks to months to accompany the patient, in an acute liver failure setting transplant teams must assess patients over days or even hours and decide on transplant eligibility. The urgent way in which these patients present for treatment, including the potential for recurrent self-harm adds to the challenge of adequate psychosocial assessment. Additionally, pre-transplant depression has been associated with lower quality of life and can have a negative effect on the post-transplant prognosis [9].

## Conclusions

In adults, iron overdose typically occurs with suicidal intent. Iron overdose can have local irritative gastrointestinal effects as well as systemic toxicity like metabolic acidosis, liver failure and multi-organ failure. Initial risk assessment is mostly based on the presumed amount of elemental iron ingested, with ingestions ≥60 mg/kg body weight of elemental iron associated with a higher risk of systemic toxicity and death. Early transfer to an acute care unit of patients with concerning symptoms or larger doses ingested may be prudent as rapid deterioration of hepatic and cardiovascular functions can occur. Liver transplantation is an option in patients with fulminant liver failure, albeit with variable results.
